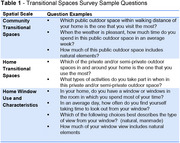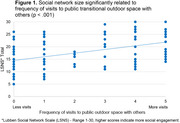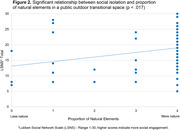# Design for Social Connection: Impact of Transitional Spaces on Older Adults with and without Dementia

**DOI:** 10.1002/alz70858_101126

**Published:** 2025-12-25

**Authors:** Diana Anderson, Emily Waskow, Tayan Zhang, Brandon E Frank, Andrew E. Budson

**Affiliations:** ^1^ Boston University School of Medicine, Boston, MA, USA; ^2^ VA Boston Healthcare System, Jamaica Plain, MA, USA; ^3^ Boston University, Boston, MA, USA; ^4^ Boston University Alzheimer's Disease Research Center, Boston, MA, USA; ^5^ Boston University Chobanian & Avedisian School of Medicine, Boston, MA, USA; ^6^ Boston Center for Memory, Newton, MA, USA

## Abstract

**Background:**

Built space is increasingly recognized to have measurable impact on mental and social health outcomes. Transitional spaces are indoor/outdoor areas in and around the home providing community connections (e.g., windows, porches, parks, etc.). Little is known about these community design characteristics and their impact on health. The goal of this study is to better understand how older adults utilize these transitional spaces, and the impact on health for those with and without dementia.

**Methods:**

Telephone surveys were remotely administered to community‐dwelling cognitively healthy older adults and those with early‐stage dementia. A novel built environment survey assessing various spatial scales of (1) community, and (2) home design features was administered (Table 1). Measures of loneliness, social isolation, mood, anxiety, and cognitive performance were also included. We hypothesized that greater use of residential transitional design features (e.g., windows) and greater access to outdoor spaces (e.g., parks) would be associated with positive health outcomes (e.g., less social isolation and loneliness).

**Results:**

98 older adults completed the assessment, consisting of 47 healthy adults and 51 cognitively impaired individuals, ages 50 to 90 (*M* = 73.2, *SD* = 8.0). Measures of social isolation were related to time spent in outdoor public spaces (*r* = .25, *p* = .017). Those who reported more frequent visits with others to community outdoor public spaces had significantly larger social networks (*r* = .44, *p* < .001), as did those who reported a higher proportion of natural elements in these spaces (*r* = .25, *p* = .017) (Figures 1&2). No such relationships were apparent for depression, anxiety, or loneliness (*p*s > .05). Those who frequently visited residential transitional spaces in and around the home with others also reported less social isolation (*r* = .26, *p* = .013). Additional transitional space features and their uses will be explored in further analyses.

**Conclusion:**

For older adults, home and community transitional spaces may allow ways of engaging with the social landscape and mitigate effects of isolation. Further understanding of the built environment's role in social connections can inform design strategies for improving the lives of older adults in the community.